# Trends in cigarette demand and supply in Africa

**DOI:** 10.1371/journal.pone.0202467

**Published:** 2018-08-17

**Authors:** Nicole Vellios, Hana Ross, Anne-Marie Perucic

**Affiliations:** 1 Economics of Tobacco Control Project, Southern Africa Labour and Development Research Unit, School of Economics, University of Cape Town, Cape Town, Western Cape, South Africa; 2 Department for Prevention of Noncommunicable Diseases, World Health Organization, Geneva, Switzerland; University of California San Diego School of Medicine, UNITED STATES

## Abstract

**Background:**

Since the tobacco epidemic is moving from developed to developing countries, it is important to understand trends in cigarette demand and supply. We focus on the African market since it offers the tobacco industry one of the best expansion potentials.

**Data:**

A large variety of data from commercial (Canadean, Euromonitor International, tobacco industry reports), governmental (United Nations Comtrade, national statistics), and academic sources (Cigarette Citadels Map and journal articles) were utilized.

**Methods:**

We compile data from multiple sources to study cigarette demand in Africa from 1990 to 2012. We then focus on cigarette production and international cigarette trade to detect structural changes in cigarette supply over the past few decades. We contrast data from these different sources.

**Results:**

Cigarette demand and supply data in Africa is limited and is sometimes inconsistent across different sources. Given this caveat, we found that the overall demand for cigarettes (measured by sales) in the 22 countries covered by Canadean, which represents 80% of Africa’s population, increased by 44% (from 165.6 billion cigarettes to 238.5 billion cigarettes) from 1990 to 2012. This higher demand has been met by cigarette production increasing in these 22 countries by 106% during the same period. As a result, Africa has moved from being a net importer to a net exporter of cigarettes. At the same time, cigarette production has become more concentrated as the tobacco industry has strategically identified certain countries as production hubs.

**Conclusions:**

Monitoring the production, consumption and trade of cigarettes by improving the quality of surveillance is necessary to understand the demand and supply of cigarettes not only in Africa, but globally.

## Introduction

While cigarette consumption is decreasing in most developed countries, it is increasing in many developing countries, where markets are often unregulated, cigarette prices are low, and tobacco control laws are weak or not enforced.[1] Well aware of this situation, the tobacco industry aggressively promotes its products in low- and middle-income countries to attract new consumers.

Over the past 25 years the global tobacco market has become increasingly concentrated owing to reductions in barriers to trade, foreign direct investment, privatization of State Owned Tobacco Companies (SOTC), and a wave of mergers and acquisitions.[2] The trend is towards further concentration of the four Transnational Tobacco Companies (TTC), which are among the world’s largest and most productive firms, with ample ability to restructure their production processes to suit the changing situation.[3] Concentration in the tobacco industry has resulted in substantial consolidation, to such an extent that the four TTCs and one state-run company in China controlled 85% of the global tobacco market in 2014.[2] This agency power of TTCs has been a key driver of tobacco industry globalization.[3]

Africa is in a particularly vulnerable position since the majority of its countries are still in the early stages of the tobacco epidemic. Only recently have several African countries begun to monitor the epidemic by conducting reliable surveys [4, 5]. Three countries (Cabo Verde, South Africa and Uganda) have recent, representative and periodic data for both adults and youth and seven (Cameroon, Comoros, Kenya, Mauritius, Senegal, Seychelles, and Togo) have recent and representative data for both adults and youth.[6]

To capture the scope of the tobacco epidemic on the continent, we examine cigarette market trends in Africa by describing trends in both the demand and supply of cigarettes using various data sources. We compare data sources for cigarette sales, production and trade. We consider the locations of cigarette production facilities in Africa and how they change over time as the tobacco industry selects certain countries as production hubs.

## Data and methods

We use a large variety of data from commercial (Canadean, Euromonitor International, tobacco industry reports), governmental (United Nations Comtrade, World Bank, national statistics) and academic sources (the Cigarette Citadels Map and journal articles). These were complemented with Google searches to ensure that no important sources were missed.

Euromonitor International and Canadean (previously ERC) are both market research firms. Euromonitor International provides data and information on production, trade, sales, brands and the competitive landscape of cigarettes in eight African countries (Algeria, Cameroon, Egypt, Kenya, Morocco, Nigeria, South Africa, Tunisia) for the years 2000–2014. Euromonitor International sources data from official statistics, trade associations, company reports, trade press and trade interviews. Canadean covers the same eight countries as Euromonitor International, plus an additional 14 countries (Angola, DRC, Ethiopia, Ghana, Ivory Coast, Libya, Madagascar, Mauritius, Mozambique, Sudan, South Sudan, Senegal, Tanzania, Zambia and Zimbabwe). Canadean provides similar data as Euromonitor for 1990–2012 for all 22 countries, covering about 80% of Africa’s population.[7] Canadean states that it compiles its data from a range of sources including United Nations (UN) Comtrade, national trade statistics, US Department of Agriculture and the tobacco industry.

Comparing Euromonitor International trade data to Canadean data was limited by the fact that Euromonitor International only has trade data from 2000 for a subset of countries covered by Canadean.

In an attempt to cross-verify Canadean and Euromonitor data with official sources, we located sales and production data for Kenya (available from DataFirst [8], original source: Kenyan National Bureau of Statistics) and sales data for South Africa from various sources: Statistics South Africa, Auditor-General, Republic of South Africa and the Tobacco Board.

The Cigarette Citadels Map, a project based at Stanford University, locates cigarette factories using an interactive global map that tags factories across the world.[9] Each tag contains information (to various degrees) about an individual tobacco factory, including links to the company’s website (where available). Country location of some production facilities is also identified in Euromonitor International, Canadean and tobacco industry reports.

UN Comtrade contains official trade statistics on cigarette exports, imports, re-exports and re-imports for different time periods, as reported to the UN by both exporting and importing countries.

We searched Google with the following search terms:

- “tobacco production” and “Africa”;
- “cigarette production” and “Africa”;
- “cigarette manufacturing” and “Africa”;
- “cigarette manufacturing” and “(country name)”; and
- “Africa/(country name)” and “cigarette factory”

Tobacco industry annual reports and webpages retrieved from this search were useful for locating additional production facilities (Table 1). We consulted news articles and, in the cases of South Africa and Kenya, we were successful in cross-referencing information from government websites.

**Table 1:** Locations of production facilities by manufacturer as of May 2017.

**Imperial Tobacco** (IT) (Total: 12)	**Burkina Faso** [17, 18] (two factories)[9] **Central African Republic** [9, 17, 18] **Chad** [17, 18] (two factories)[9] **Congo** [17, 18] (called ‘Societe Industrielle Agricole du Tabac Tropical’)[9] **Ivory Coast** [9, 17–19] **Madagascar** [9, 17, 18] **Mali** [9, 17, 18] **Morocco** [9, 17, 18] **Senegal** [9, 17, 18] **Algeria** [9, 19]
**British American Tobacco** (BAT) (Total: 8)	**South Africa** [9, 16, 20] **Mozambique** [9, 21] **Kenya** [9, 22] **Eritrea** [9, 15] **Algeria** [9, 23, 24] **Nigeria** [9, 16, 25, 26] **Togo** [16] **Ivory Coast** [16]
**Japan Tobacco International** (JTI) (Total: 5)	**South Africa** [9, 27, 28] **Tanzania** [9, 27, 29] **Tunisia** [27] **Sudan** [27] **Nigeria** [30]
**Phillip Morris International** (PMI) (Total: 2)	**Senegal** [9, 31, 32] **Algeria** [24, 31]–STAEM is a joint venture between PMI and a consortium of UEM businessmen
**State-owned Tobacco Companies** (SOTC) (Total: 10)	**Algeria** [24, 33] Société Nationale des Tabacs et Allumettes (SNTA) has four production facilities,[9, 24] Société des Tabacs Algéro-Emiratie (STAEM) **Tunisia** [33, 34]—Régie Nationale des Tabacs et des Allumettes (RNTA) and Manufacture des Tabacs de Kairouan (MTK)[34] **Libya** [33] **Egypt**–Eastern Company SAE [33, 35, 36] (2 factories) [9] Produces a number of brands under license for BAT, PMI and JTI [35]
**Others** (Total: 25)	**Botswana**–Benson Craig [9, 37] **South Africa**–Gold Leaf Tobacco,[9, 38] Amalgamated Tobacco Manufacturing,[39] United Africa Tobacco Manufacturing,[40] Mastermind Tobacco SA,[28] Gallaher SA,[28] Carnilinx Tobacco Company,[41] Folha Manufacturers [42] **Zimbabwe**–Savanna, [9, 43] Breco [9, 44] **Morocco**–La Société Marocaines des Tabacs, [45] new Emirati manufacturer (manufacturing PMI brands) [45] **Kenya**–Cut Tobacco [46] **Angola**–Barco Trading [9, 47] **Mozambique**–Sonil (Sociedade do Niassa) [9, 48] **Malawi**–Nyasa Manufacturing Company [9, 49] **Zambia**–Roland Imperial Tobacco [9, 50] **Seychelles**–Amalgamated tobacco Company [9, 51] **Uganda**–Leaf Tobacco & Commodities [9, 52] **Ethiopia**–National Tobacco Enterprise [9, 53] **Sudan**–Afrah Tobacco [9, 54] **Cape Verde**–Sociedade Caboverdiana de Tobacos [9, 55] **Libya**–General Tobacco Company [9, 56] **Tunisia**–Régie Nationale des Tabacs et des Allumettes (RNTA) (2 factories)[9, 57]

In the end, we primarily relied on the Canadean data for sales, trade and production data because of both its wide geographical coverage and the length of its time series. We calculated per capita cigarette sales for those aged 15+ using Canadean consumption data and World Bank population data.[7]

## Results

**Fig 1:**
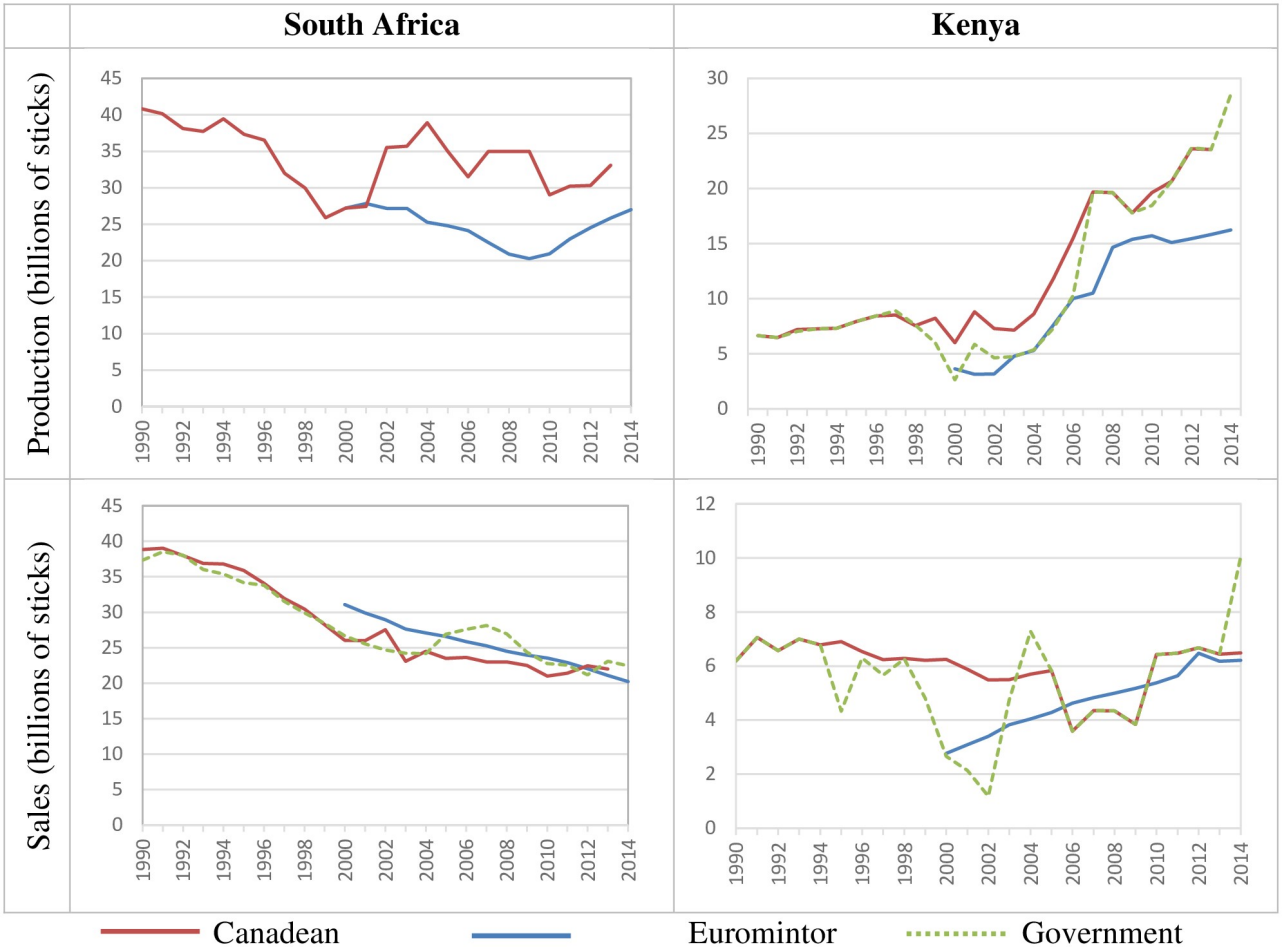
Production and sales of cigarettes in South Africa and Kenya (1990–2014). Source: Sales data for South Africa from Statistics South Africa (1998), Auditor-General (selected years), Republic of South Africa (selected years) and Tobacco Board (selected years); production and sales data for Kenya available from DataFirst [8].

Data on cigarette consumption, production, and trade in Africa is very limited and in some cases, the data sources are contradictory. Fig 1 presents four graphs for South Africa and Kenya showing estimates for production and sales from three different data sources: Canadean, Euromonitor International (from 2000) and government sources (except for cigarette production estimates for South Africa, which we were unable to find). Although the Canadean and Euromonitor production estimates for 2000 in South Africa coincide (27,196 billion), Euromonitor reports lower production for all other years. From 2009 to 2014, Euromonitor reports a 33% increase in production. Sales statistics for South Africa reported by all three data sources are quite close and exhibit similar trends even though Euromonitor consistently reports higher sales than Canadean does, with the exception of 2012 and 2013.

For Kenya, the production estimates from the three data sources are generally similar with some data points being identical. However, Canadean and government sources show the rise in production to be steeper than Euromonitor does. There is more variation across the data sources for cigarette sales in Kenya, although Government and Canadean data coincide for years 2005–2013.

Compared to Canadean, Euromonitor consistently reports lower total exports and imports by volume for the period 2001–2012 for the eight countries covered by both datasets. These two data sources only marginally agree on import volume for 3 years (2004–2006), for which Euromonitor reports an average imported volume of 11.8 billion sticks per year, while Canadean reports 14.3 billion sticks per year. Both data sources agree that exports from these eight countries have increased over time, but Canadean reports this increase at a much higher rate. For example, for 2012 Euromonitor claims that the total cigarette export from these eight countries amounted to 12.9 billion cigarettes, while Canadean estimates the same export to have reached 41.7 billion sticks. Comtrade’s comparable statistics fall between the two estimates, with 25.4 billion sticks exported in 2012.

Despite these data inconsistencies, we were able to make several observations regarding trends in the demand and supply of cigarettes in Africa.

**Fig 2:**
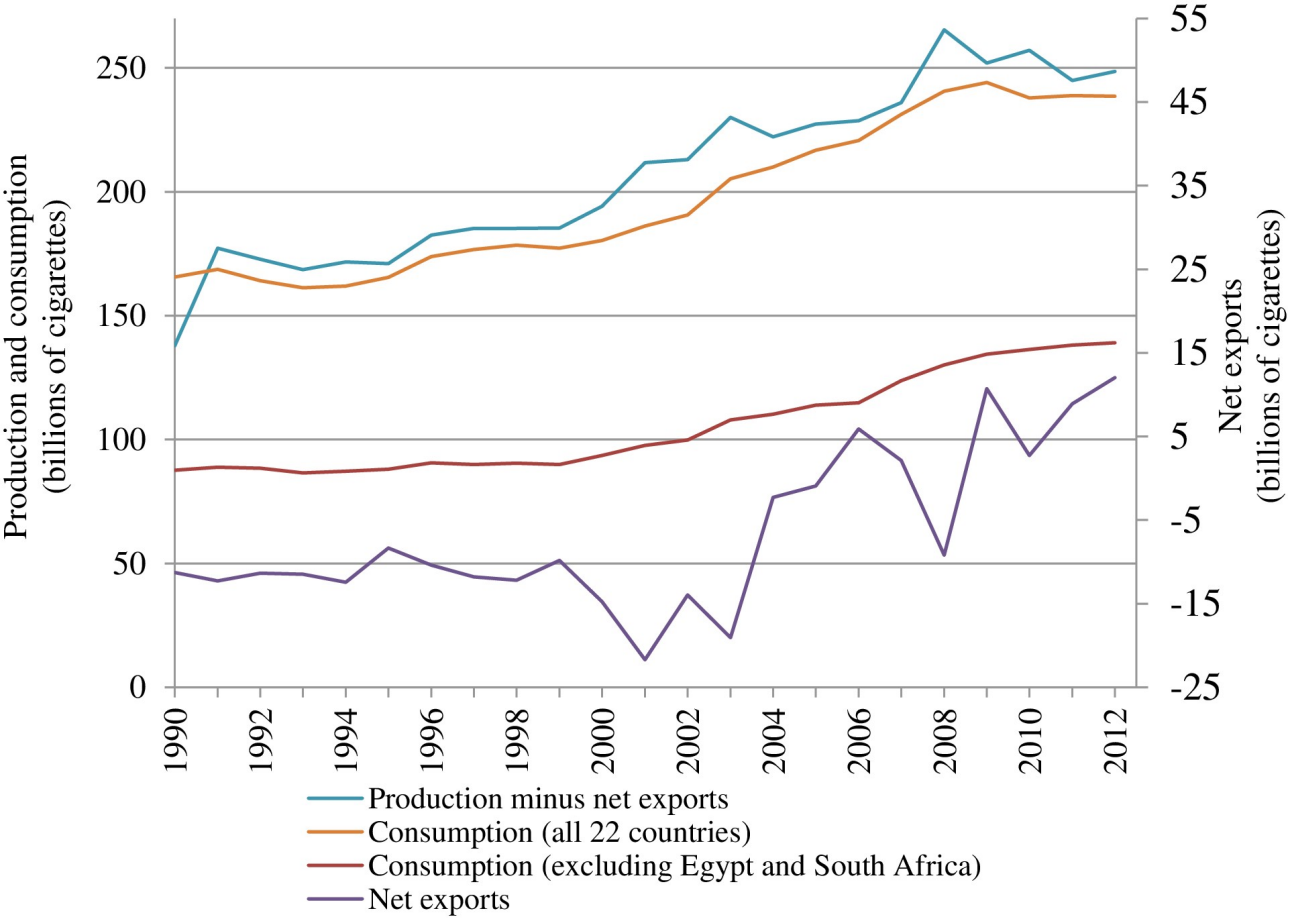
Cigarette consumption, net exports and production less net exports, 1990–2012. Source: Canadean. Countries include Algeria, Angola, Cameroon, DRC, Egypt, Ethiopia, Ghana, Ivory Coast, Kenya, Libya, Madagascar, Mauritius, Morocco, Mozambique, Nigeria, North and South Sudan, Senegal, South Africa, Tanzania, Tunisia, Zambia and Zimbabwe.

Cigarette consumption (measured by sales) in the 22 African countries covered by Canadean has been increasing rapidly over the past two decades (Fig 2). Between 1990 and 2012, cigarette consumption increased from 165.6 billion to 238.5 billion cigarettes, or by 44%. The slight decrease in cigarette consumption in 2010 is driven by Egypt where cigarette excise tax increased by 87% in that year, causing the average cigarette retail price to increase by about 44%.[10] Given the significant role of Egypt and South Africa in the cigarette market on the continent, we also present cigarette consumption in the remaining 20 countries excluding Egypt and South Africa. The cigarette consumption on this subset of countries increased even faster—by 59% between 1990 and 2012.

Production, less net exports based on Canadean data (Fig 2) is a measure of the quantity of cigarettes that remain in a country for local consumption and should be equal to domestic sales. However, this consumption measure is consistently about 6% higher than domestic sales. The difference could be due to factors such as undeclared sales, pilferage or the distribution of free samples.

As production increased over the past two decades, Africa has moved from being a net importer of cigarettes to a net cigarette exporter. Higher production is now able to satisfy the increasing cigarette demand in Africa.[11]

Even though the total cigarette demand in Africa seems to be primarily driven by population growth (between 1990 and 2012, the population (15+) in the 22 countries increased by 86%), many countries report increasing smoking rates. Although current smoking prevalence among females increased in only six out of 42 countries in the AFRO region between 2000 and 2010, current smoking prevalence among males increased in 25 out of 40 countries. [12]

**Fig 3:**
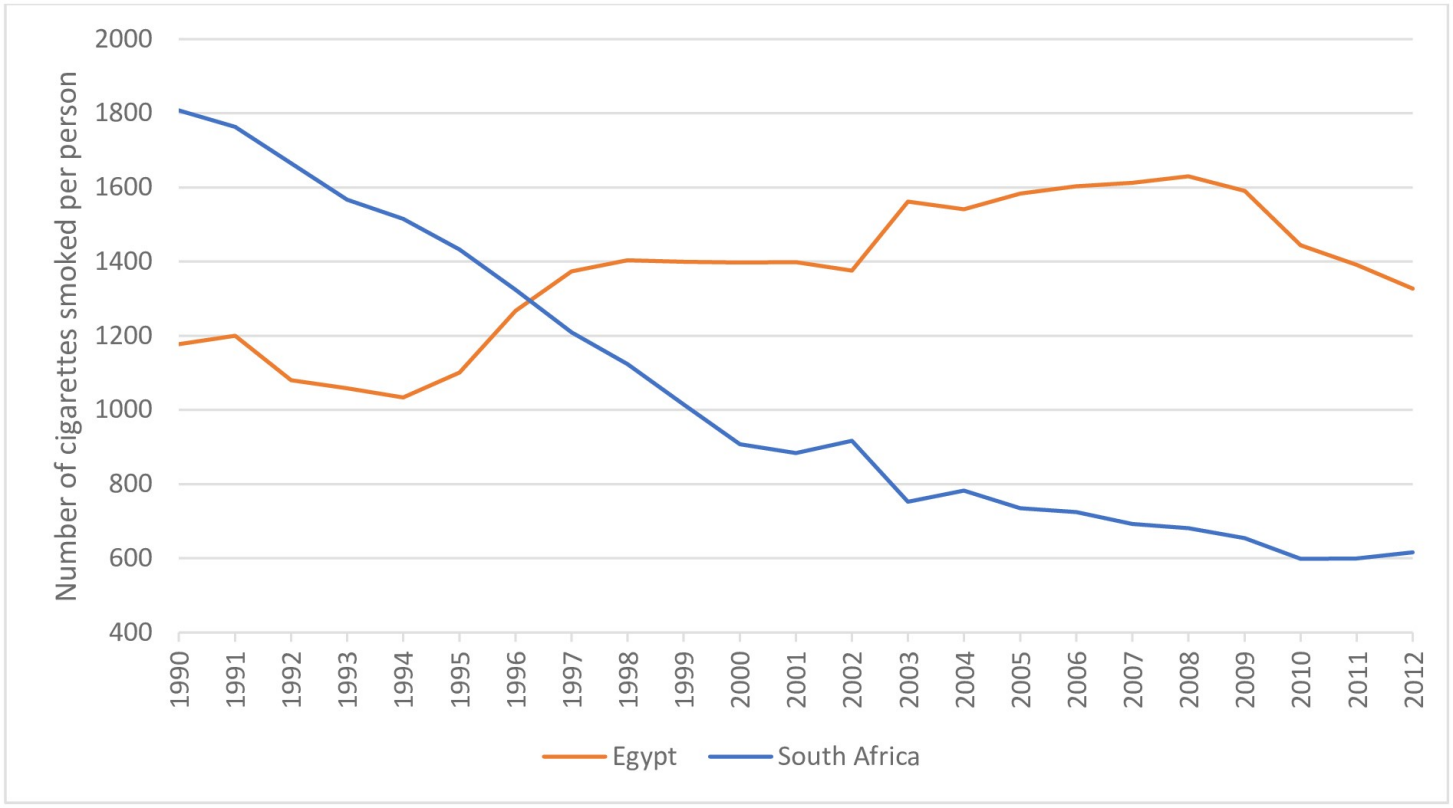
Number of cigarettes smoked per person per year (age 15+). Source: Canadean and World Bank population data.

A substantial body of literature has conclusively shown that there is an inverse relationship between tobacco prices and tobacco consumption. While other policies (such as advertising bans and smoke free areas) are effective tobacco control measures, excise taxes are the most effective. South African and Egyptian markets responded to effective tobacco control measures implemented in 1995 and 2010 respectively. Between 1995 and 2012, per capita cigarette consumption decreased by 57% in South Africa and by 8% in Egypt between 2010 and 2012 (Fig 3).

On the supply side, we identified 62 production facilities located in 30 African countries in 2017 (Table 1). Philip Morris International (PMI), Japan Tobacco International (JTI), Imperial Tobacco (IT) and British Tobacco own 27 of these production facilities, 10 are State-owned Tobacco Companies (SOTC) and the remaining 25 belong to other smaller manufacturers (Table 1). We do not list an additional seven factories that are on the Cigarette Citadels Map as those facilities were not verified by a second source or the company’s own website. We found that some of them were closed. For example, BAT factories in Ghana and the DRC were closed in 2006 and 2014, respectively.[13–15] The 2016 Euromonitor International Global Company Profile [16] also incorrectly reports on the BAT factory in Ghana.

**Fig 4:**
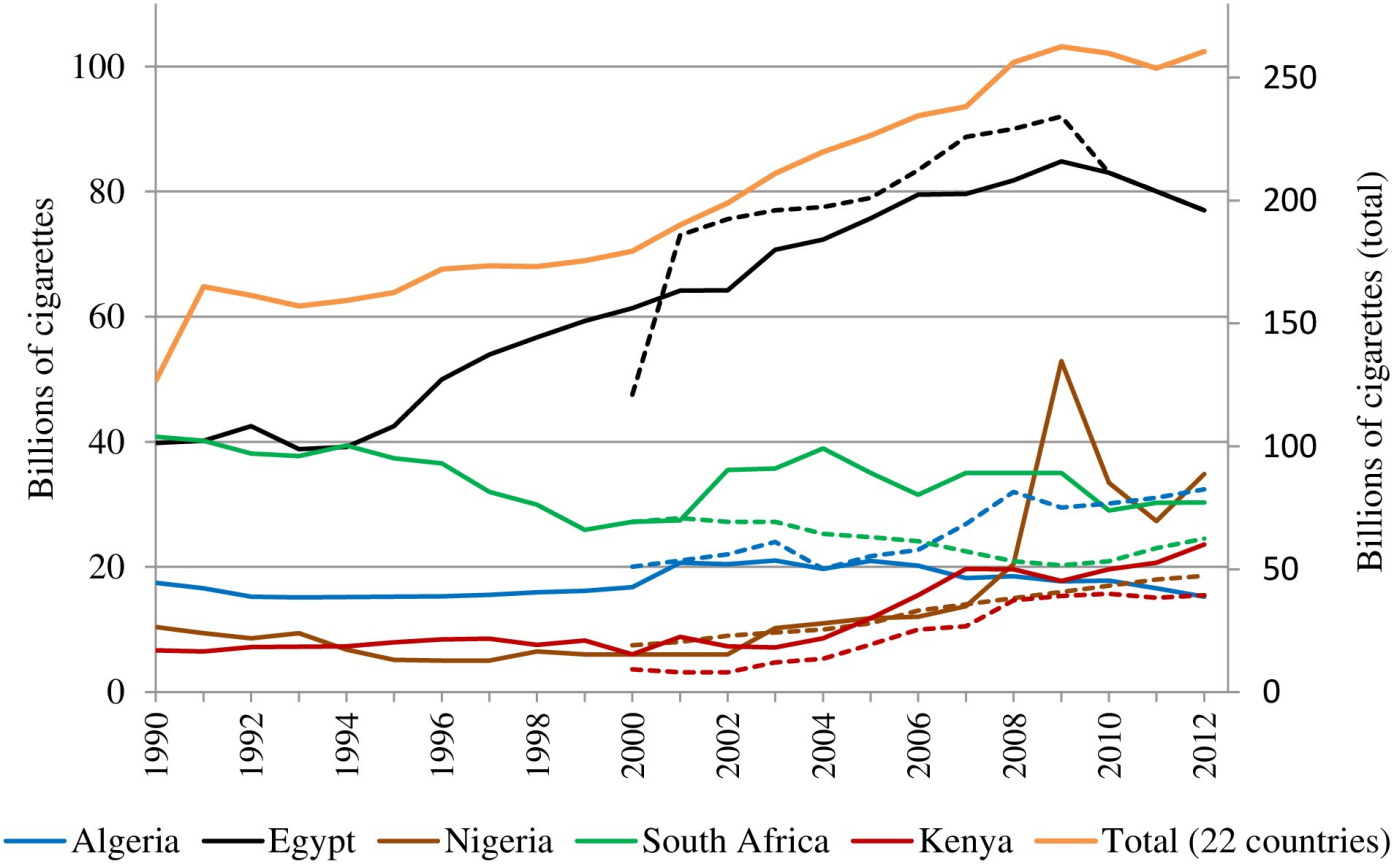
Cigarette production in Africa 1990–2012: The main producers (primary axis) and the total production (secondary axis). Source: Canadean (solid lines) and Euromonitor International (dashed lines).

Between 1990 and 2012, cigarette production in the 22 countries covered by Canadean increased by 106% (Fig 4: orange line; secondary axis). The most important production hubs have been established in Algeria, Egypt, Kenya, Morocco, Nigeria, South Africa and Tunisia.

In early 1990s, cigarette production in Africa was dominated by Egypt and South Africa with each country producing about 40 billion sticks per year.[58, 59] Since 1994, the two countries developed differently: cigarette production in South Africa began to decline but took a fast upward trajectory in Egypt, making the country the leading cigarette producer in Africa. According to Canadean, the production of cigarettes in Egypt more than doubled between 1993 and 2009 (38.8 billion pieces to 84.8 billion pieces). This supply has been substantially absorbed by an increase in domestic cigarette sales of 130% during this period (from 37.9 billion to 87.1 billion). Although Egypt is the main producer in Africa, it is not a major exporter since most of its production is consumed locally.[60] Egypt imports only a small quantity of cigarettes owing to its high cigarette import duty [60] that favours the state-controlled monopoly Eastern Company SAE.[61] Eastern Company SAE operates eight main manufacturing plants and 380 distribution warehouses across Egypt.[36] Brands of other companies are produced under licence by Eastern Company SAE.[61]

Even though it was on a level with Egypt in 1994, by 2012 South Africa manufactured fewer than half of the number of cigarettes as Egypt. The main cigarette manufacturer in South Africa, BAT South Africa, produces around 20 billion sticks of cigarettes annually for both local consumption (BAT brands account for about 70% of consumption in South Africa) and for export,[62] but its production is declining since the closure of its main production facility in Paarl in 2008.[16, 63] While Euromonitor International (2015) states that most of the cigarettes produced for export in South Africa are exported to European and American markets, 2014 Comtrade data shows that more than 80% of exports from South Africa were destined for African markets.[62]

Apart from Egypt and South Africa, other main cigarette producers in Africa are Nigeria, Algeria and Kenya. BAT Nigeria (BATN) serves both the local and international markets, with one factory producing for the domestic market only, and another that functions as an export base.[25] BATN’s objective is to produce enough cigarettes to meet domestic demand, without any need for imports.[25]

State-owned Société Nationale Des Tabacs & Allumettes (SNTA) is the dominant cigarette manufacturer in Algeria. SNTA has five cigarette producing facilities, primarily supplying the domestic market.[24] Prior to 2005, the Algerian market was closed to foreign suppliers, but in 2005 SNTA created a joint venture called Société des Tabacs Algéro-Emiratie (STAEM) with a consortium of UAE businessmen and began production in 2009. STAEM manufactures Philip Morris, Japan Tobacco and Imperial Tobacco brands in their local facilities.[64]

The two main tobacco companies in Kenya, the locally owned Mastermind and the transnational British American Tobacco Kenya (BATK), use Kenya as a regional production base, supplying both local and neighbouring regional markets in at least 17 countries.[46, 65–67] As a result, most cigarettes produced in Kenya are exported.[68, 69] By 2015, BATK was the largest producer of cigarettes in East and Central Africa.[69] The vast majority of cigarettes consumed in Kenya are produced locally.[46] To develop Kenya as a regional manufacturing centre, both Mastermind and BATK closed factories in other countries: Mastermind closed factories in Uganda and Tanzania,[66] while BAT closed factories in Zambia,[70] Uganda,[46] Mauritius,[46] Rwanda,[46] Cameroon[71] and the DRC.[13] (Table 2). Column 4 of Table 2 demonstrates the impact of these closures on domestic imports. For example, cigarette imports to Mauritius more than tripled between 2005 and 2007.

**Table 2:** Production concentration case study—BAT in Africa.

Country and data of closure of BAT factory	Total imports, year prior to factory closure, million cigarettes (main import partner)	Total imports, year after factory closure, million cigarettes (main import partner)
Uganda (2006) [46]	42 (Kenya)	2 192 (Kenya)
Ghana (2006) [72]	14 (UAE)	912 (South Africa)
Mauritius (2006) [46]	407 (China)	1 236 (Kenya)
Zambia (2006) [70]	27 (South Africa)	1 189 (Kenya)
Rwanda (2006) [46]	0.3 (China)	520 (Kenya)
Cameroon (2007) [71]	687 (Gabon)	2 576 (South Africa)
South Africa (2008) [16, 63]	1 307 (China)	1 479 (China)
DRC (2014) [13]	(No data)	(No data)

Source: Import data (column 2 & 3) from UN Comtrade.

Even though TTCs have been consolidating their production in order to save costs, they use the threat of factory closures to influence decision-makers to introduce weaker tobacco control policies.[2] Most recently, BAT threatened to close its Heidelberg factory in South Africa if the government introduced plain packaging for cigarette packs.[73]

## Discussion

In order to monitor the tobacco epidemic emerging in Africa, there is an urgent need for reliable country-level data, in accordance with Article 20.2 of the WHO’s Framework Convention on Tobacco Control, which encourages the parties to the convention to integrate tobacco surveillance programs into national health surveillance programs.[74] Frequent and reliable data will support research efforts aimed at influencing policy outcomes.

Although the data we used has limitations, we were able to note several trends. The overall demand for cigarettes (measured by sales) in Africa has increased from 165.6 billion cigarettes to 238.5 billion cigarettes (44%) from 1990 to 2012. At the same time, cigarette production in Africa has grown so that it is able both to meet this higher demand and to export to other continents. We identified 62 cigarette production facilities in Africa that belong to TTCs, (44%), SOTCs (16%) and other smaller producers (40%).

Production growth and the restructuring of production facilities in Africa has resulted in industry consolidation, where five cigarette production hubs operate in Egypt, South Africa, Niggeria, Kenya and Algeria. Another potential production hub is Ethiopia, where JTI purchased 40% shares of the Ethiopia’s National Tobacco Enterprise for half a billion dollars in 2016.[75] The cigarette markets in Algeria and Egypt are dominated by SOTCs. As a result, governments of these countries have vested interests in supporting the tobacco industry, which could harm tobacco control efforts.

Future research should explore the market shares of TTCs, SOTCs and other smaller producers in Africa, and how these are changing over time, in order to study how the African market is adapting to higher cigarette demand and the globalisation of the tobacco industry.

## Conclusion

African governments need to collect and report reliable production, sales and trade data. This can be achieved by improving surveillance systems with the help of new technologies. To avoid further increases in consumption, African governments should implement and enforce policies such as higher excise taxes, advertising bans, smoke-free areas and warning labels as outlined by the World Health Organization’s Framework Convention on Tobacco Control.[76]

**Limitations**: We had to rely on commercial data due to the lack of official statistics. Since the information on production facilities in Africa is limited, it is possible that we have omitted some of them and/or that some of those identified have closed.
